# Precise sizing of aortic valvular leaflet reconstruction using 4-dimensional computerized tomography: A personalized approach

**DOI:** 10.1016/j.xjtc.2024.08.018

**Published:** 2024-09-07

**Authors:** Hani K. Najm, Batool Barodi, John P. Costello, Munir Ahmad, Lama Dakik, Justin T. Tretter

**Affiliations:** a Division of Pediatric Cardiac Surgery, Valve Procedural Planning Center, Cleveland Clinic Children's and the Heart, Vascular, and Thoracic Institute, Cleveland Clinic, Cleveland, Ohio; b Department of Pediatric Cardiology, Valve Procedural Planning Center, Cleveland Clinic Children’s and the Heart, Vascular, and Thoracic Institute, Cleveland Clinic, Cleveland, Ohio

**Figure:**
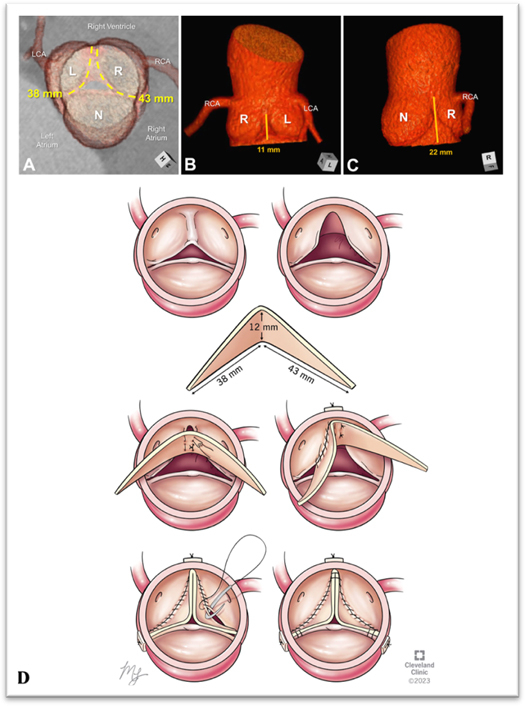
CT 3D reconstruction of the patient's bileaflet aortic valve and root by creating a butterfly patch.

**Central Message**

Advanced imaging gives a precise and personalized approach to leaflet extension that results in improved initial aortic valve competency and may improve long-term function of the aortic valve.

Bicuspid aortic valve (BAV) is the most common congenital cardiac abnormality.^1^ Aortic valve (AV) replacement with prosthetic valves is fraught with morbidity and late mortality.^2^ AV repair is an effective strategy that avoids such disadvantages.^2^, ^3^, ^4^ Predictability of BAV repair for severe regurgitation is technically demanding, with several techniques employed yielding variable success. In a functional BAV with fusion between 2 of the leaflets, <20% of cases will present with very asymmetric commissural orientation (120°-139°), which presents more challenging BAV anatomy for repair.^5^ Most described techniques depend on only a few preoperative measurements but rely heavily on intraoperative measurement of leaflet geometry. The goal is to reconstruct a competent trileaflet or BAV with maximized durability.

We present a case of bileaflet extension creating symmetrical tricuspidization of a severely regurgitant functional BAV with trisinuate aortic root (AoR) and very asymmetrical commissures. Precise dimensions of the leaflet extension were obtained by preoperative 4-dimensional (4D) computed tomography. This approach allowed preplanned measurements of AoR and valvar geometry, and the leaflet coaptation's measures, which were utilized in personalizing leaflet reconstruction. Written consent was obtained from the patient's legal guardian, and our institutional review board waived the need for review given the de-identified information.

## Case Presentation

**Figure 1:**
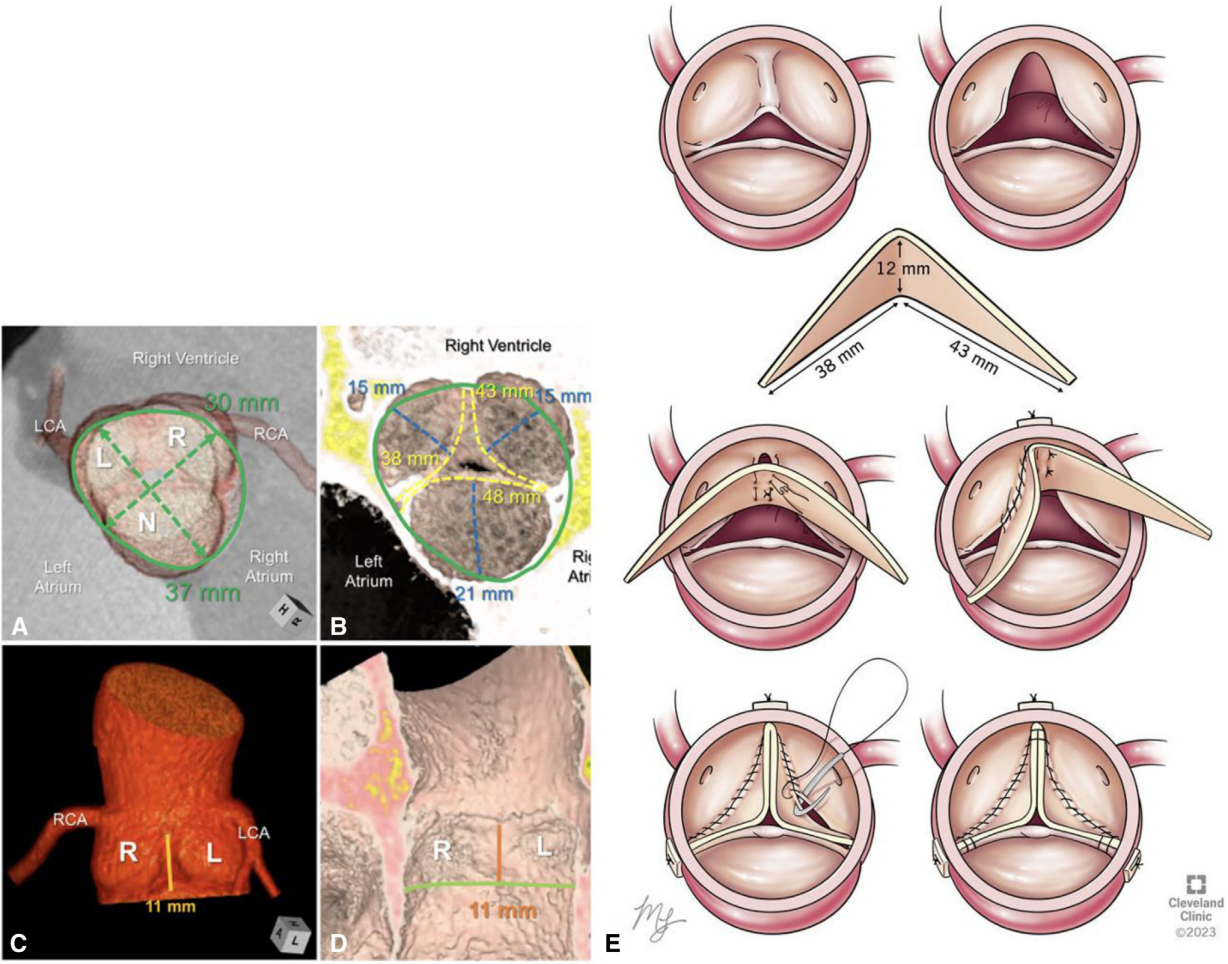
Computed tomographic 3-dimensional reconstructions of the functionally bileaflet aortic valve and trisinuate aortic root depict its pertinent measurements. A, The virtual basal ring (*green oval line*) and its major and minor axis measurements (*hashed double-headed arrows with adjacent measurements*) are depicted relative to the overlying sinuses of the aortic root. B, The geometric heights (*blue hashed lines*) and free margin lengths (*yellow hashed lines*) of each leaflet are depicted, assessing the free margin lengths of the fused coronary leaflets as if they were surgically divided. C and D, The hypoplastic commissural height between the fused leaflets (*orange line*) is half the height of the normal commissures. E, Drawing of the butterfly patch that was created using the measurements demonstrated in Panels A through D. *LCA*, Left coronary artery; *RCA*, right coronary artery; *L*, left coronary sinus or leaflet; *R*, right coronary sinus or leaflet; *N*, noncoronary sinus or leaflet.

**Figure 2:**
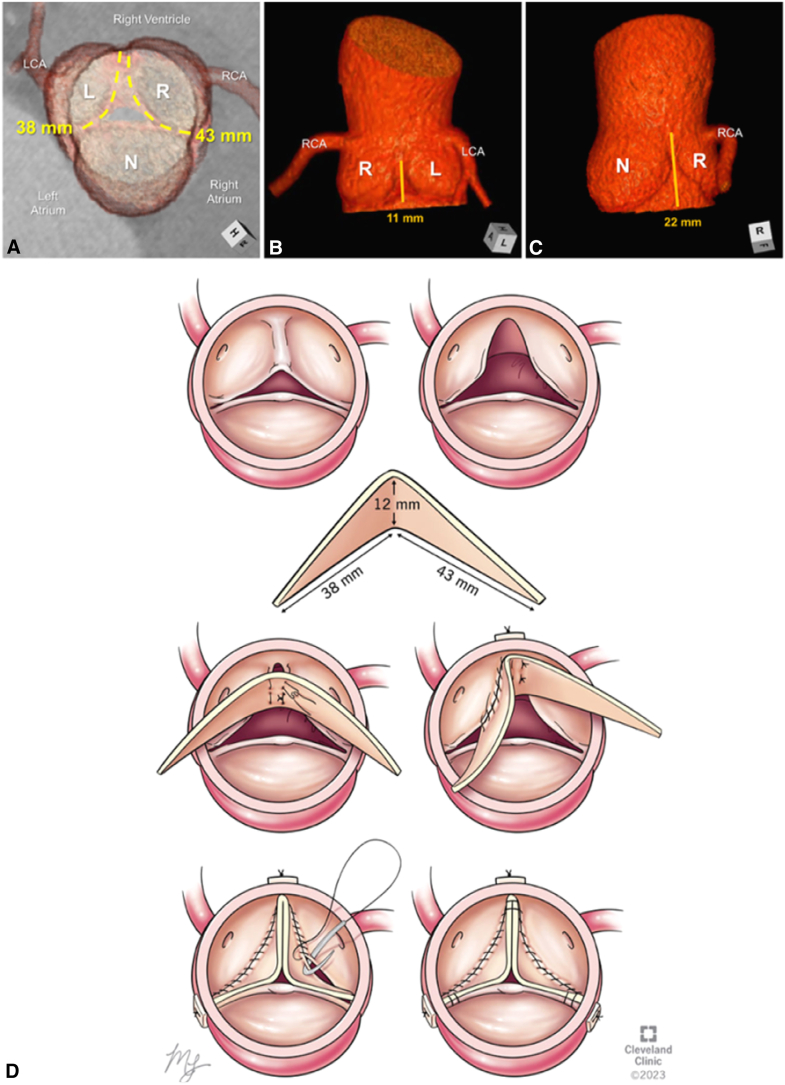
Computed tomography 3-dimensional reconstruction of the patient's bileaflet aortic valve; A, demonstrates the free margin length of leaflets, B, depicts the shortened commissure height between coronary leaflets, C, depicts the normal commissure height, D, graphical rendering of the butterfly patch sizing that corresponds to the deficient leaflets and techniques of implantation. *LCA*, Left coronary artery; *RCA*, right coronary artery; *L*, left coronary sinus or leaflet; *R*, right coronary sinus or leaflet; *N*, noncoronary sinus or leaflet.

**Figure E1:**
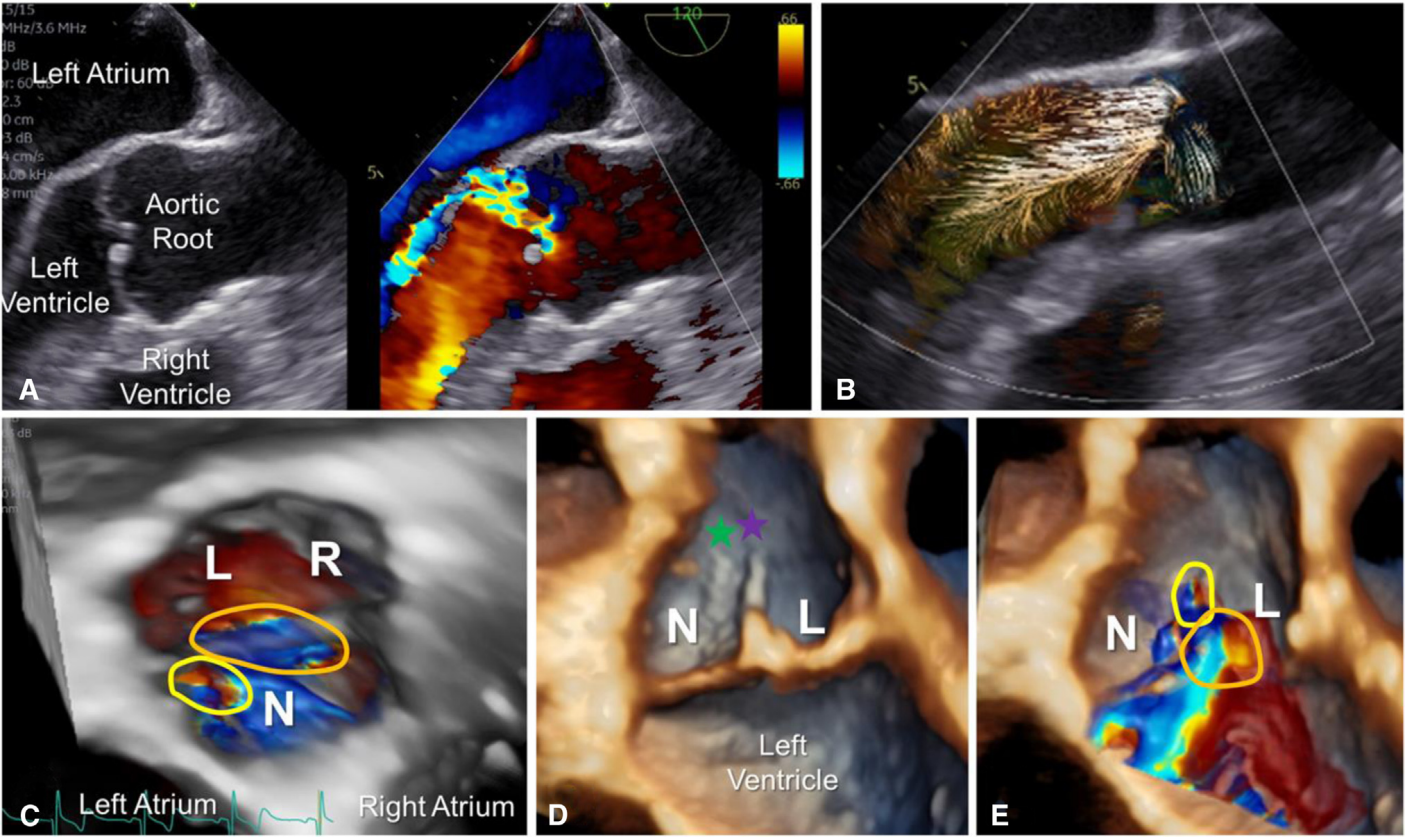
Preoperative transesophageal echocardiographic assessment. A, Color comparison of the long axis of the aortic root in diastole demonstrates severe aortic regurgitation with a large jet centrally, and second jet more posteriorly. B, Blood speckle imaging during systole across the aortic valve in long axis in the same view as Panel B demonstrates flow vectors converging at the valve leaflet tips and then directed anteriorly toward the anterior wall of the proximal ascending aorta. C, Three-dimensional color Doppler short-axis view of the aortic valve in diastole demonstrates a larger central regurgitant jet at the free edge of the fused coronary leaflets (*outlined in orange*) with a second smaller jet at the left (L) and non(N)-coronary leaflet commissure. D, Three-dimensional long-axis view of the aortic root of this commissure during diastole demonstrates malalignment between these 2 leaflets at their commissure (*green star demonstrates the superior attachment of the N; purple start demonstrates the superior attachment of the L*) creating an obvious coaptation deficiency. E, Corresponding 3-dimensional color Doppler confirms this commissural coaptation deficiency (*outlined in yellow*), separate from the central coaptation deficiency (*outlined in orange*). *R*, Right coronary leaflet.

An 18-year-old man presented with severe aortic regurgitation (AR), left ventricular dilation, and functional BAV with fusion between the coronary leaflets and trisinuate AoR. A 4D computed tomography scan demonstrated fused coronary leaflets with very asymmetric commissural position (135°) favoring a tricuspidization approach. There was moderate annular (3.8 × 3.0 cm), mild root (3.7 cm), and mild sinotubular junction dilation (3.4 cm). The intercoronary commissural height was hypoplastic (11 mm) compared with the 2 normal commissures (22-23 mm). The free margin length for left and right leaflets was 38 and 43 mm, respectively (Figure 1). A tailored butterfly patch extension of both leaflets with height of 12 mm at the base was made to elevate commissural height to match normal commissures. The length was equivalent to the free margin length of the coronary leaflets (Figure 2). Preoperative transesophageal echocardiography (TEE) confirmed these findings, revealing an additional regurgitation jet at left and noncoronary leaflet commissure related to commissural malalignment (Figure E1).

## Operative Procedure

After sternotomy, a piece of pericardium was harvested and treated with 0.6% glutaraldehyde for 10 minutes, followed by 3 6-minute rinses in normal saline. Cardiopulmonary bypass was initiated, and dissection around the AoR and coronary arteries was performed before aortic clamping. Blood cardioplegia via a retrograde coronary catheter was administered. A series of 2-0 Ti-Cron pledgeted sutures (Medtronic) passed at level of the virtual basal ring below the nadir of the leaflet from inside to outside.

The right and left leaflets were divided to the hypoplastic commissure followed by augmentation of both leaflets with the prefashioned butterfly patch of tanned autologous pericardium. Patch suturing was started at the neocommissure extension, 12 mm above the previous hypoplastic commissure by series of horizontal mattress sutures passed from inside the aorta and tied on the outside to elevate the total neocommissural height to equal the other 2 normal commissural heights. Each wing of the patch was sutured to the superior aspect of the corresponding coronary leaflet (Figure 1, *E*, and Video 1). An additional figure-of-8 commissuroplasty of the left and noncoronary commissures was performed to address the identified commissural coaptation deficiency (Figure E1, *C*-*E*). Two rings of #30 polyethylene terephtalate tube graft were cut, divided, and passed below the coronary buttons externally. Ti-Cron sutures were passed through this strip and tied down to create a subcoronary annuloplasty. One suture in the noncoronary sinus was used to join 2 ends. Aortic closure above the commissures was reinforced with a piece of bovine pericardium to stabilize the sinotubular junction and augment suture line hemostasis.

**Figure E2:**
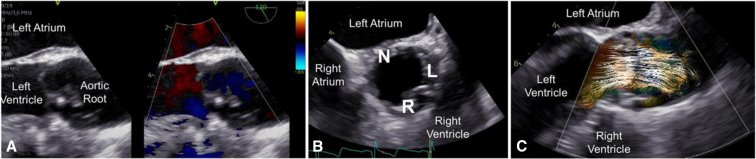
Postoperative transesophageal echocardiographic assessment. A, Color comparison of the long axis of the aortic root in diastole demonstrates no visible aortic regurgitation. B, Short axis of the aortic valve in systole demonstrates improved leaflet mobility. C, Blood speckle imaging during systole across the aortic valve in long axis in the same view as Panel A demonstrates laminar flow vectors directed into the long axis of the ascending aorta, markedly improved when compared with the preoperative assessment. *N*, Noncoronary leaflet; *L*, left coronary leaflet; *R*, right coronary leaflet.

Cardiopulmonary bypass was weaned with excellent hemodynamic parameters. Postoperative TEE demonstrated trace AR and no aortic stenosis with normal systolic function with improved flow dynamics as seen by blood speckle imaging (Figure E2).

## Postoperative Status

The postoperative course was unremarkable, and our patient was discharged home on day 4. TEE before discharge showed reduced left ventricle size with trace AR. Leaflet effective heights normalized from 4 mm preoperatively to 11 mm postoperatively. At the last follow-up echocardiograph, 20 months postsurgery, there was mild AR with peak gradient of 16 mm Hg.

## Discussion

We describe a surgical repair technique of severely regurgitant AV guided by precise geometrical assessment of the AoR and AV. Our novel approach leverages the high spatial resolution of 4D computed tomography to provide a personalized blueprint for repairing the complex anatomy of a congenitally malformed AoR and restoring valvular competency.^6^ We believe utilizing preoperative personalized measurements of the needed leaflet extension tissue in loaded conditions is far superior to the intraoperative measurement currently utilized. In particular in young patients with pliable tissues, intraoperative measurements could be misleading, resulting in less optimal early and late results. With this in mind, we now routinely obtain cardiac computed tomography for our patients undergoing surgery for congenital AoR and/or valvular disease to quantitatively assess the geometry of the AoR and its valve.^6^

Replacing deficient leaflet tissue with autologous pericardial tissue has many benefits. First, it creates symmetric trileaflet valve with significant coaptation area that persists despite AoR growth. Second, the size of autologous pericardium needed for 2-leaflet construction is limited; therefore, it can be available despite the redo nature of these operations. Finally, replacing a BAV with a pulmonary autograft graft could subject the patient to lifelong conduit replacements and autograft failure.

## Conclusions

Although future interventions may be required, creation of a large coaptation area has the potential to delay reoperation. Despite the challenging BAV anatomy experienced with very asymmetrical commissures, the patient's successful course can be attributed to this 4D imaging-based approach guiding surgical planning.

## Conflict of Interest Statement

The authors reported no conflicts of interest.

The *Journal* policy requires editors and reviewers to disclose conflicts of interest and to decline handling or reviewing manuscripts for which they may have a conflict of interest. The editors and reviewers of this article have no conflicts of interest.

## Supplementary Data

Video 1 Advanced imaging-based reconstruction of bileaflet aortic valve and trisinuate aortic root using 4D CT. Measurements guided butterfly patch creation and precise sizing of aortic valve leaflet extension. Video available at: https://www.jtcvs.org/article/S2666-2507(24)00352-3/fulltext.
