# Supplementary figures and images for: Precise sizing of aortic valvular leaflet reconstruction using 4-dimensional computerized tomography: A personalized approach
Source: JTCVS Tech. 2024 Sep 7;28:115–9. doi: 10.1016/j.xjtc.2024.08.018 (PMC11632312; doi:10.1016/j.xjtc.2024.08.018)

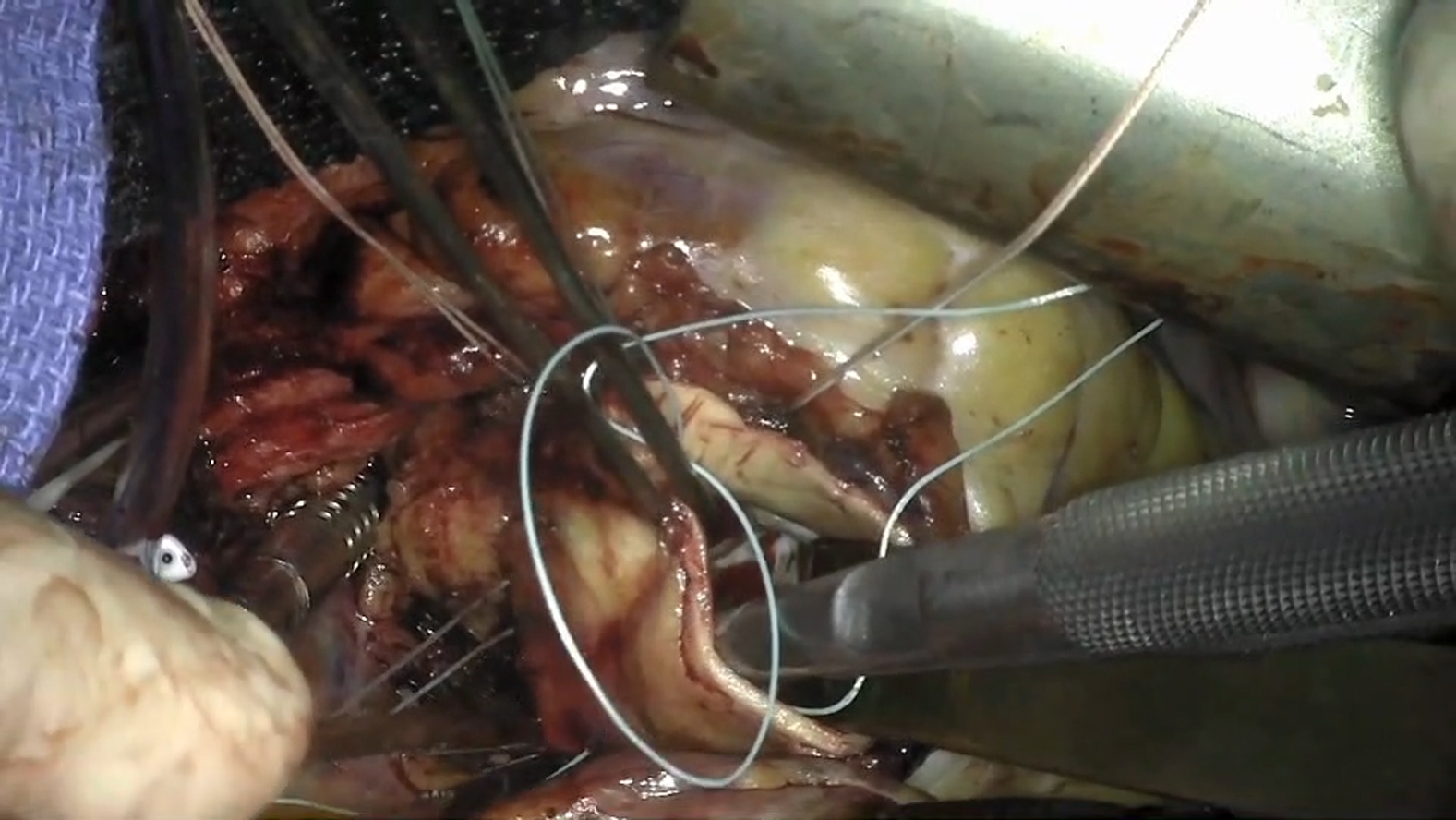

Supplement: Video 1 — Advanced imaging-based reconstruction of bileaflet aortic valve and trisinuate aortic root using 4D CT. Measurements guided butterfly patch creation and precise sizing of aortic valve leaflet extension. Video available at: https://www.jtcvs.org/article/S2666-2507(24)00352-3/fulltext. [file fx2.jpg]
